# Fecal microbiota transplantation and short-chain fatty acids reduce sepsis mortality by remodeling antibiotic-induced gut microbiota disturbances

**DOI:** 10.3389/fimmu.2022.1063543

**Published:** 2023-01-11

**Authors:** Xiran Lou, Jinfang Xue, Ruifei Shao, Yan Yang, Deyuan Ning, Chunyan Mo, Fuping Wang, Guobing Chen

**Affiliations:** ^1^ Medical School, Kunming University of Science and Technology, Kunming, China; ^2^ Department of Emergency Medicine, The First People’s Hospital of Yunnan Province, Kunming, China

**Keywords:** sepsis, gut microbiota, antibiotic, fecal microbiota transplantation, short-chain fatty acids

## Abstract

**Objective:**

Sepsis is the leading cause of death in critically ill patients. The gastrointestinal tract has long been thought to play an important role in the pathophysiology of sepsis. Antibiotic therapy can reduce a patient’s commensal bacterial population and raise their risk of developing subsequent illnesses, where gut microbiota dysbiosis may be a key factor.

**Methods:**

In this study, we analyzed the 16S rRNA of fecal samples from both healthy people and patients with sepsis to determine if alterations in gut bacteria are associated with sepsis. Then, we developed a mouse model of sepsis using cecal ligation and puncture (CLP) in order to examine the effects of fecal microbiota transplantation (FMT) and short-chain fatty acids (SCFAs) on survival rate, systemic inflammatory response, gut microbiota, and mucosal barrier function.

**Results:**

Sepsis patients’ gut microbiota composition significantly differed from that of healthy people. At the phylum level, the amount of Proteobacteria in the intestinal flora of sepsis patients was much larger than that of the control group, whereas the number of Firmicutes was significantly lower. Mice with gut microbiota disorders (ANC group) were found to have an elevated risk of death, inflammation, and organ failure as compared to CLP mice. However, all of these could be reversed by FMT and SCFAs. FMT and SCFAs could regulate the abundance of bacteria such as Firmicutes, Proteobacteria, Escherichia Shigella, and Lactobacillus, restoring them to levels comparable to those of healthy mice. In addition, they increased the expression of the Occludin protein in the colon of mice with sepsis, downregulated the expression of the NLRP3 and GSDMD-N proteins, and reduced the release of the inflammatory factors IL-1β and IL-18 to inhibit cell pyroptosis, ultimately playing a protective role in sepsis.

**Disccusion:**

FMT and SCFAs provide a microbe-related survival benefit in a mouse model of sepsis, suggesting that they may be a viable treatment for sepsis.

## 1 Introduction

Sepsis is a life-threatening organ failure caused by a dysregulated host response to infection and is responsible for approximately 20% of deaths worldwide (1, 2). It has become a prevalent problem among intensive care unit (ICU) patients and a popular reason for ICU admission. Since the outbreak of coronavirus disease 2019 (COVID-19), many severely unwell or critically ill COVID-19 patients have demonstrated severe metabolic acidosis, decreased liver and renal function, and severe lung injury (3–5). According to the International Consensus, these patients met the diagnostic criteria for sepsis and septic shock and shared common pathophysiological mechanisms of multiorgan injury with bacterial sepsis (6). Presently, there have been significant advances in understanding sepsis’s pathophysiology. However, antibiotics, intensive fluid resuscitation, vasoactive agents, surgical therapy, and supportive care are currently the only options for treating sepsis. Targeted treatments for sepsis are currently not available (6). The number of people who get sepsis and die from it continues to rise, and its treatment remains a global problem.

The gut microbiota, comprising bacteria, viruses, fungi, protozoa, and archaea, contributes to the host’s lifelong health by playing essential roles in the growth and functioning of the host immune system and the intestinal epithelial barrier (7). The gut microbiota has been shown to play an important role in both health and illness (8). In fact, disturbed gut microbiota lost their protective function after infection or injury and even exacerbated organ damage and failure (9). Recent research suggests that an altered intestinal microecology may play a significant role in the etiology and progression of sepsis (10, 11). Therefore, it is crucial to understand the relationship between intestinal flora and sepsis.

Patients with sepsis are usually treated with antibiotics. A study found that about 75% of ICU patients received antibiotics daily (12). Long-term use of broad-spectrum antibiotics might inhibit sensitive microorganisms, produce more drug-resistant bacteria, and eventually lead to intestinal flora disorders, aggravating the disease (13). The epidemiological study indicated that antibiotic use increases intestine bacterial translocation, which may increase vulnerability to systemic infection (14). However, antibiotics are currently used to treat sepsis (15), so we hypothesize that the overuse of antibiotics in sepsis patients may result in intestine microecological dysfunction, hence increasing sepsis patient mortality. In the early stages of critical illness, more than half of the commensal microbiota may be lost, making it almost impossible to replenish gut microbiota diversity (16, 17). Fecal microbiota transplantation (FMT) is the process of giving a healthy person’s feces, which contain thousands of bacterial communities, to someone who is sick. This helps to replace the commensal bacteria that have been killed off and may steer the microbiota of the patient toward a healthier state. Studies have shown that the use of FMT or probiotics to change the gut microbiota can help protect sepsis patients (18). Short-chain fatty acids (SCFAs) consisting of two to six carbons are crucial to the health of the intestinal lining. SCFAs are predominantly made up of acetate, propionate, and butyrate, which are produced by gut bacteria during the digestion of dietary fiber (19). SCFAs are critical compounds that promote intestinal integrity. Several studies have reported a decrease in SCFAs concentration in sepsis patients, suggesting that this reduction may influence the systemic inflammatory response after severe injury in patients (20, 21). Maintaining the focus on SCFAs may be crucial for sepsis patient care in the ICU. Therefore, further research into the connection between gut bacteria metabolites and disease could lead to the development of novel therapeutic options for sepsis patients.

Pyroptosis is a type of programmed cell death that involves the release of cellular contents and proinflammatory cytokines. It is an important part of the innate immune response that protects against pathogens and microbial infections (22). It involves inflammasome priming, NOD-like receptor protein-3 (NLRP3) inflammasome assembly and activation, gasdermin D (GSDMD) cleavage and pore creation, and proinflammatory molecule release (23). Once pyroptosis is overexpressed, the inflammatory response is triggered in the surrounding cells and tissues, exacerbating the injury and ultimately resulting in organ failure or septic shock due to a systemic inflammatory response (24). In sepsis models, there is substantial evidence that neutrophils undergo pyroptosis and that interleukin 18 (IL-18) and interleukin 1 β (IL-1β) levels are elevated (25, 26).

In this study, we did a 16S rRNA sequencing analysis of the fecal sample of sepsis patients to look for changes in their gut microbiota. In addition, by constructing a mouse model of sepsis, we intended to determine if FMT or SCFAs can attenuate sepsis and the corresponding underlying mechanism. We discovered that antibiotics alter the makeup of normal mice’s intestinal flora, and that FMT and SCFAs can ameliorate the disorder of intestinal flora following antibiotics and lower the mortality of septic mice. Our research may help in the search for a new way to treat sepsis-related gut microbiota disruption.

## 2 Materials and methods

### 2.1 Clinical sample information

This study included a control group (n = 12) and a group with sepsis (n = 22). The control group had no previous septic illness or trauma. Individuals who had taken antibiotics or probiotics within the previous eight weeks were excluded. We collected fecal samples from 22 sepsis patients in the Intensive Care Unit of the First People’s Hospital of Yunnan Province between December 2020 and December 2021. All patients who met the inclusion criteria were consecutively enrolled during the study. Patients met Sepsis-3 criteria established by the Society of Critical Care Medicine/European Society of Intensive Care Medicine (SCCM/ESICM) (27). In addition, a set of exclusion criteria existed: Under the age of 18, pregnancy, organ transplantation, long-term immunosuppression, malignancy, hepatitis virus infection, or chronic renal insufficiency are contraindications. All sepsis patients accepted basic antibiotic therapy, which included cefoperazone tazobactam, meropenem, and piperacillin-tazobactam. The local medical ethics commission authorized every experiment (Project No: KHLL2021-036). Both patients and healthy volunteers provided their informed consent in writing.

### 2.2 Experimental procedures

This study followed National Institutes of Health guidelines and was approved by Kunming Medical University’s Research Ethics Committee (Project No: KMMU20221600).

### 2.3 Animal experiment

Male C57BL/6 mice weighing 20 ± 2 g were housed in pathogen-free cages at the Experimental Animal Center of Kunming Medical University at a temperature of 22 ± 1°C and relative humidity of 65 ± 5% (both supplied by the Department of Zoology, Kunming Medical University). During the experiment, mice were kept in cages with a 12 h light/dark cycle and free access to water and food. Mice were randomly separated into five groups after a week of adaptive feeding: Sham group (sham operation), CLP group (CLP-induced sepsis model), ANC group (antibiotics+normal saline+CLP), AFC group (antibiotics+FMT+CLP), and ASC group (antibiotics+SCFAs+CLP). Mice were gavaged with 0.2 ml broad-spectrum antibiotic mixture (vancomycin 1.5 g/70kg and imipenem 2 g/70kg, purchased from Solarbio company, Beijing, China) once daily for four days to disturb the gut flora. Next, mice in the ANC group were gavaged 0.3 ml of normal saline once daily for three days; mice in the AFC group were gavaged 0.3 ml of the fecal bacterial solution once daily for three days; and mice in the ASC group were gavaged 0.3 ml of a mixture of short-chain fatty acids (Sigma, USA). The physiological relevance of this SCFA mixture (67.5 mM acetate, 25.9 mM propionate, and 40 mM butyrate) has been shown (28). On day seven, we subjected all mice to the CLP-induced sepsis model and sacrificed them 16 h following the CLP or sham operation, collecting the necessary samples for further molecular biology experiments. The onset of sepsis was triggered by cecal ligation and puncture (CLP). Pentobarbital sodium (50 mg/kg) is used to induce anesthesia in animals prior to the CLP procedure. Under aseptic settings, a 1.5 cm incision was made in the lower abdomen to expose the cecum. One centimeter of the distal cecum was ligated fully with a 3.0 silk suture, punctured once with an 18-gauge needle, and then put back into the peritoneal cavity. After that, the incisions in the skin and peritoneal wall were closed with sutures. After surgery, mice received a 1 ml sterile saline (0.9% solution) peritoneal injection. The Sham group underwent cecal exposure and abdominal incisions, but no ligation or puncture was performed. Mice had access to food and drink after the procedure.

### 2.4 FMT assay

We obtained fecal bacterial solution from healthy male C57BL/6 mice ranging in weight from 18 to 22 g, whose diet consisted of regular laboratory food and tap water. Following the guideline, each person should donate 30 g of feces, which should then be diluted with 3-5 times as much sterile saline (for example, 30 g of feces diluted with 150 ml of normal saline) (29). Prepare fecal bacterial solution according to relevant literature. The specific steps are as follows: (1) using the method of stimulating mouse anus, collect the fresh feces of healthy mice and put them into EP tube; (2) Dilute, weigh 0.2 g feces, put it into the mixer, add 1ml sterile normal saline, stir and mix evenly; (3) Centrifugation: centrifuge the suspension at 2000 rpm for 5 min in a 15 ml centrifuge tube, collect the supernatant, and repeat the process three times; (4) By gavage, the new bacterial solution was sub packed in 1.5 ml EP tube. The AFC group mice were given 0.3 ml fecal bacterial solution for three days. After three days, mice were subjected to CLP and sacrificed 16 h later when tissues were collected.

### 2.5 Bacterial counts

To evaluate the aerobic bacterial load in the peritoneal cavity, we lavaged the peritoneal cavity with 5 ml of sterile saline. Log serial dilutions were performed. A portion (500 ul) of each dilution was then plated on blood agar plates (20210714B, BioMe’rieux, Shanghai, China) and incubated under aerobic conditions at 37°C for 24 h. After plating serial dilutions of lavage fluid on blood agar, total bacterial counts were estimated based on the dilution factor and number of bacterial colonies. The results were represented as colony-forming units per milliliter (CFUs/mL).

### 2.6 Histological analysis and immunohistochemistry

Mice were sacrificed 16 h following CLP surgery, and their colon, lung, liver, and kidney tissues were collected and fixed in 4% paraformaldehyde for 24 h before being embedded in paraffin. 5-μm tissue sections were stained with H&E and viewed under a light microscope for observation. Antigens were retrieved for immunohistochemistry by treating samples for 10 min at 98°C in 10 mM citrate buffer (Beyotime, Shanghai, China). Endogenous peroxidase was inhibited with 10% H_2_O_2_ for 20 min and nonspecific antigens were inhibited with sera for 30 min at room temperature prior to immunohistochemical examination. At 4°C for 12 h, the slides were treated with particular primary antibodies. Antibodies utilized included Occludin (1:250, 66378-1-lg, proteintech, USA). Secondary antibodies conjugated with HRP were then applied to the slides.

### 2.7 Enzyme-linked immunosorbent assays (ELISA)

All animals were anesthetized with 50 mg/kg of pentobarbital sodium before their orbital venous blood was extracted. The blood was centrifuged at 3000 rpm for 15 min and 4°C to separate the serum for subsequent use. Serum levels of diamine oxidase (DAO), D-lactate and IL-1β were measured by ELISA kit (MEIMIAN, Jiangsu, China).

### 2.8 Western blot

The mice were administered a strong anesthetic and slaughtered 16 h following the CLP procedure. The intestinal samples were swiftly collected and frozen at -80°C for future use. The samples were homogenized with RIPA lysis buffer (Beyotime Biotechnology, Shanghai, China) at 12, 000 xg and 4°C for 20 min. To generate the standard curve, the supernatant was treated with a Bbicinchonininc acid (BCA) kit (Beyotime, Shanghai, China) and the absorbance was measured at 570 nm. The sample protein concentration was maintained at 10 g/l. Equal amounts of protein (20 mg) were separated by 10% SDS-PAGE and transferred to PVDF membranes (Invitrogen, USA) from several models. The membranes were blocked for 1 h at room temperature in blocking solution (5% BSA in TBST), then incubated overnight at 4°C with NLRP3 (1:1000, Abcam, USA), GSDMD (1:1000, Abcam, USA), GSDMD-N (1:1000, Cell Signaling, USA), IL-18 (1:1500, Abcam, USA), and beta-actin (1:5000, Cell Signaling, USA) antibodies. The sample was then treated with an IgG secondary antibody coupled with horseradish peroxidase for 1 h at room temperature. Using the ChemiDoc XRS technology, the protein bands were generated and photographed (Bio-Rad, USA). Using Image J, the relative density of the blots was measured.

### 2.9 Transmission electron microscopy (TEM)

For TEM, two 0.5×0.5 cm mini-segments of intestinal tissue from each group were removed and put in a fixative at 4°C for 2-4 h. The segments were rinsed three times for 15 min in 0.1 M PBS, postfixed in 1% osmium tetroxide in PBS, dehydrated in a graduated series of ethanol solution, then implanted in an oven at 60°C for 48 h. Using uranyl acetate and lead citrate, samples were cut into 60-80 nm sections and stained. Electronic microscopy was used to evaluate the sections (JEM-1400Flash, Japan).

### 2.10 16S rRNA diversity sequencing

Fecal samples were collected from colon tissues and immediately stored at -80°C. The Mag-Bind Soil DNA kit (Omega, M5635-02, USA) was used to extract microbial DNA from stools according to the manufacturer’s instructions. The V3-V4 hypervariable region was then analyzed for 16S rRNA amplicons using primer pairs 338F (59-ACTCCTACGGGAGGCAGCAG-39) and 806R. (59-GGACTACHVGGGTWTCTAAT-39). The Illumina MiSeq system was used for sequencing per the manufacturer’s instructions (Illumina MiSeq, USA). Using Research software, the effective tags were clustered into operational taxonomic units (OTUs) with 97% similarity (version 11.0.667). For bioinformatics analysis, Mothur and QIIME2.0 were utilized as software. Shannon, Simpson, Chao, and ACE indices were utilized to assess alpha diversity. PCoA was utilized to examine the beta diversity based on Bray-Curtis distance, and Adonis was used to test the P-value. Using microbial taxonomy, a comparison of sample composition and an examination of particular community variations between groups were conducted.

### 2.11 Metabolomics analysis

The -80°C refrigerator-stored samples were thawed on ice. A 20 mg sample was added to a 400 l solution (Methanol: Water = 7:3, V/V) containing an internal standard and vortexed for 3 min. The material was sonicated for 10 min in an ice bath, vortexed for 1 min, and then frozen for 30 min at -20°C. The sample was centrifuged at 12,000 rpm for 10 min at a temperature of 4°C. The sediment was then removed, and the supernatant was centrifuged at 12,000 rpm for 3 min at 4°C. For LC-MS analysis, 200 l aliquots of supernatant were transferred. The LC-MS system acquired all samples following machine orders. The analytical conditions were as follows, UPLC: column, Waters ACQUITY UPLC HSS T3 C18 (1.8 µm, 2.1 mm*100 mm); column temperature, 40°C; flow rate, 0.4 mL/min; injection volume, 2 μL; solvent system, water (0.1% formic acid): acetonitrile (0.1% formic acid); gradient program, 95:5 V/V at 0 min, 10:90 V/V at 11.0 min, 10:90 V/V at 12.0 min, 95:5 V/V at 12.1 min, 95:5 V/V at 14.0 min.

### 2.12 Statistical analysis

SPSS 20.0 was used to conduct statistical analysis. All experimental information was presented as mean ± SD. To investigate between-group differences, an unpaired Student’s t-test (normally distributed) or Mann–Whitney U test (not normally distributed) was employed. Multiple group comparisons utilizing the ANOVA and LSD tests. For the ANOVA testing, *post-hoc* analysis was performed. P < 0.05 was considered statistically significant.

## 3 Results

### 3.1 Patients with sepsis have a disruption in gut microbial composition

In this study, there were 12 healthy volunteers and 22 sepsis patients. The cohort’s mean SD age was 53.00 ± 21.46. Sepsis patients had an average APACHE II score of 18.18 ± 5.42. **Table 1** provides detailed demographic and clinical information for sepsis patients. In the sepsis group, serum lactate (Lac) and procalcitonin (PCT) levels were significantly higher. There were no statistically significant differences in age or gender between the groups.

**Table 1:** General characteristics of the patients cohort.

Characteristics	Sepsis group(n=22)	Control group(n=12)
Age, years	53.00 ± 21.46	43.25 ± 9.25
BMI, kg/m²	22.79 (21.38-23.91)	23.07 ± 3.75
Gender M/F	14/8	5/7
APACHE II score	18.18 ± 5.42	–
SOFA score	8.23 ± 3.22	–
Lac(mmol/l)	3.8 (2.73-6.53)	–
PCT(ng/ml)	33.25 (4.36-109.55)	–

Data with continuous variables were expressed as median values and interquartile ranges (IQR). Data based on a normal distribution were expressed as means ± SD. APACHE II, acute physiology and chronic health evaluation II; SOFA, sequential organ failure assessment; Lac, lactate; PCT, procalcitonin.

**Figure 1:**
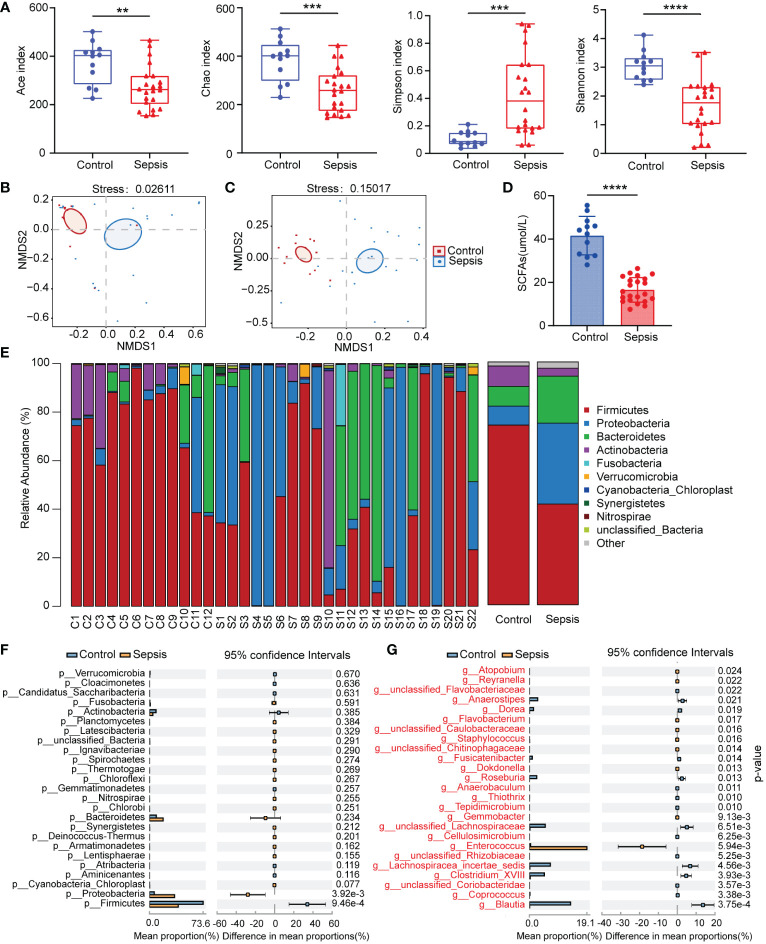
Human sepsis-related alterations in the gut microbiota. **(A)** The Ace, Chao, Shannon, and Simpson indices were used to compare the alpha diversity profiles of healthy individuals (n = 12) and patients with sepsis (n = 22). **(B, C)** NMDS analysis of beta diversity at the phylum and genus levels. Different hues symbolize different groups. The species in the two samples are more similar the closer the two sample points are to each other. **(D)** Comparison of serum SCFAs levels among two groups. **(E)** The distribution of the community at the phylum level. Different colors indicate distinct species, and the length of the columns indicates the relative abundance of those species in the group sample (represented by the Y-axis). **(F, G)** Variation in gut microbiota between the groups was statistically significant at the phylum and genus levels. **P < 0.01; ***P < 0.001; ****P < 0.0001.

We analyzed the 16S rRNA of fecal samples from both healthy people and patients with sepsis to determine if alterations in gut bacteria are associated with sepsis. The Ace, Chao, Shannon, and Simpson indices were calculated for groups to evaluate the within-sample α-diversity and were shown to characterize the richness and variety of bacteria. The sepsis group had a significantly lower α-diversity as compared to the control group (**Figure 1A**). The NMDS plot based on Bray-Curtis dissimilarity across samples demonstrated that the flora composition of sepsis patients differed significantly from that of healthy control (**Figures 1B, C**). In patients with sepsis, there is a dramatic drop in serum SCFAs (**Figure 1D**). The relative abundances of all samples’ phylum levels were presented (**Figures 1E, F**). At the phylum level, Proteobacteria and Bacteroidetes were more abundant in the sepsis group than in the control, but Firmicutes were less abundant in the sepsis group. At the genus level, we found even greater variation between patients than we saw at the phylum level in the bacterial makeup we analyzed. Patients with sepsis had a significantly greater intestinal *Enterococcus* abundance compared to healthy controls (**Figure 1G**). These findings showed that sepsis patients’ gut microbiota composition significantly differed from that of healthy people. Critically ill people have a bad microbiome full of disease-causing pathobionts. Pathogenic Proteobacteria predominate, whereas commensal species like Firmicutes disappear.

### 3.2 FMT increased the survival rate and bacterial clearance of sepsis mice after the intervention of antibiotics

**Figure 2:**
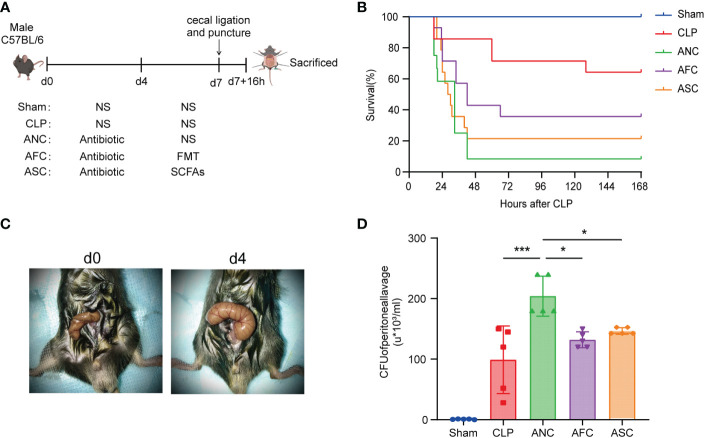
Fecal microbiota transplantation (FMT) and supplementation with short-chain fatty acids (SCFAs) attenuate CLP-induced sepsis. **(A)** Illustration of the experimental design in schematic form. **(B)** FMT and SCFAs improve survival following cecal ligation and puncture (CLP). n = 14 mice per group. Mice in the AFC and ASC groups had a higher survival rate than those in the ANC group. **(C)** A representative cecum before and after four days of antibiotic treatment. **(D)** FMT and SCFAs increased bacterial clearance in sepsis mice. *P < 0.05; ***P < 0.001.

We conducted experiments in animals to better understand the function of the gut microbiota in sepsis. Mice of the wild-type strain were administered broad-spectrum antibiotics (imipenem and vancomycin) for four days to disrupt the gut microbiota, followed by FMT and SCFAs for three days to restore a portion of the gut microbiota. Finally, cecal ligation and puncture were used to create a sepsis model (**Figure 2A**). We discovered that mice treated with antibiotics for four days had a considerably bigger cecum and lacked wrinkles (**Figure 2B**). Seven days after sepsis modeling, the survival rates of the Sham, CLP, ANC, AFC, and ASC groups were compared. In the sham operation group, there were no fatalities. At 24 h, the CLP group had a survival rate of 85.7%, and at 7 days, it had a survival rate of 64.3%. However, the 24-h and 7-day survival rates for animals in the ANC group were 58% and 8%, respectively. In addition, animals in the ANC group displayed less activity and a poorer reaction to a stimulus compared to those in the CLP group. There was a striking improvement in survival for the animals in the AFC group; the 24-h survival rate was 71% and the 7-day survival rate was 35.7% (P < 0.05; **Figure 2C**). Getting rid of the bacteria is essential for the healing process. Therefore, we counted the number of bacteria in the peritoneal cavity and analyzed how FMT and SCFAs affected the bacterial population. At 16 h post-CLP, the microbial count in the peritoneal lavage fluid was evaluated. Colonies were counted on blood agar plates 24 h after plating. No germs were present in the Sham group. However, the number of colonies in the AFC and ASC groups was much smaller than in the ANC group. Colony-forming unit (CFU) information was shown as 10^3^ CFU/mL (**Figure 2D**).

### 3.3 FMT protects against organ damage in sepsis mice after the intervention of antibiotics

**Figure 3:**
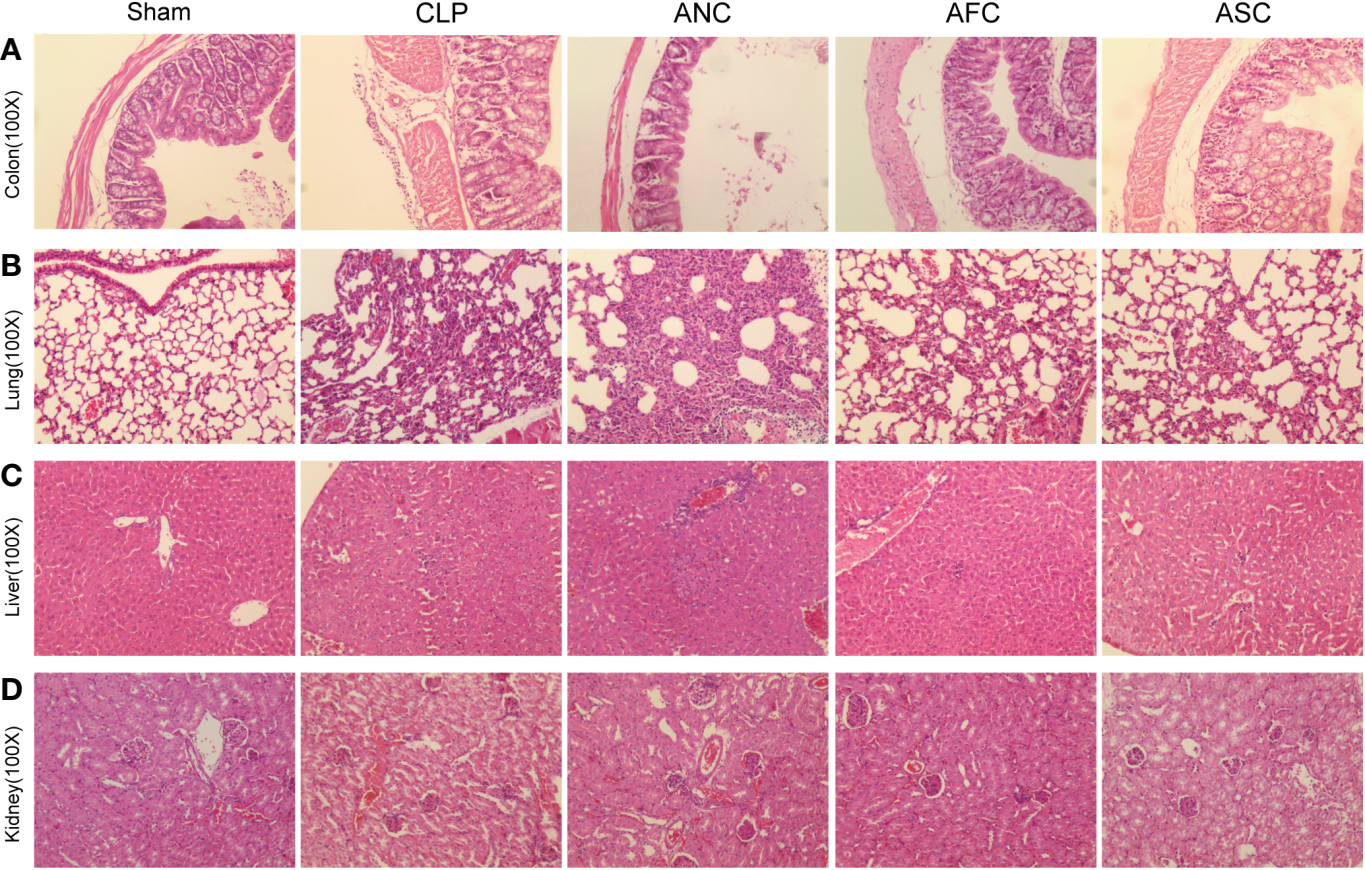
Fecal microbiota transplantation (FMT) and supplementation with short-chain fatty acids (SCFAs) prevent sepsis in mice against organ failure. **(A-D)** H&E staining revealed considerably higher levels of inflammatory infiltration, edema, and bleeding in the colon, lung, liver, and kidney tissue of the ANC group compared to the Sham group, but these abnormalities were drastically reduced in the AFC and ASC groups, originally magnification, ×100.

To investigate if the protective effect of the intestinal microbiota on sepsis extended to organ damage, we analyzed the histological sections of mice from each group after sepsis. The Sham group’s colon, lungs, liver, and kidneys had normal morphological characteristics (**Figures 3A–D**). Using hematoxylin and eosin (HE)-stained specimens of the colon, we determined that the inflammatory response and gut damage were significantly exacerbated in antibiotic-treated septic mice. Simultaneously, it was relieved in mice with sepsis treated with FMT and SCFAs. The ANC group had sparsely distributed intestinal villi and partial villus top separation (**Figure 3A**).

Images of lung tissue from the ANC group revealed that the alveolar septum had enlarged and congested, that inflammatory cells had leaked from certain alveolar cavities, and that some alveoli had merged and bullae had formed. However, administration of FMT and SCFAs attenuated alveolar septal widening, congestion, and a reduction in inflammatory cell infiltration (**Figure 3B**). Liver tissue exhibited localized necrosis and infiltration of inflammatory cells. However, compared to the ANC group, the AFC group exhibited a reduction in hepatic changes (**Figure 3C**). In renal histopathology, the ANC group exhibited morphological abnormalities in renal characteristics, including greater glomerular atrophy and sclerosis, considerably reduced number, inadequate capillary loop opening, and focal renal interstitial hemorrhage, compared to the control group. However, compared to the ANC group, the AFC group exhibited improved renal characteristics (**Figure 3D**). In conclusion, our findings indicated that treatment with FMT and SCFAs reduces the degree of systemic inflammation induced by CLP.

### 3.4 FMT protects the tight junctions in the intestinal epithelium of mice from damage caused by CLP

**Figure 4:**
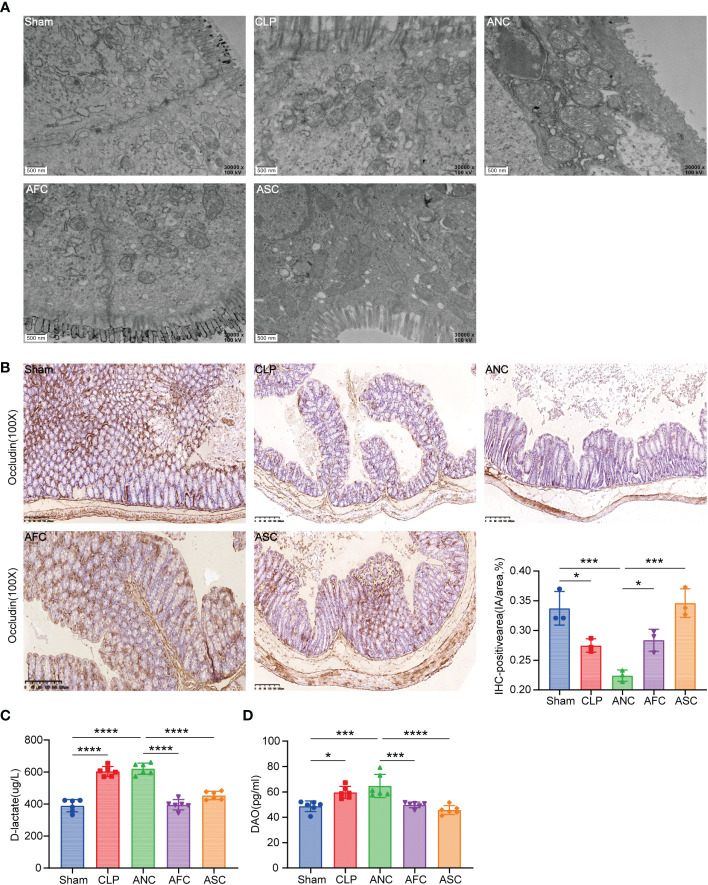
Fecal microbiota transplantation (FMT) and supplementation with short-chain fatty acids (SCFAs) protect the mouse intestinal epithelial tight junction from CLP-induced damage. **(A)** Electron micrograph of colonic segments taken from mice with different treatments. Groups are labeled as shown. Scale bar =500 nm. **(B, C)** Immunohistochemical staining of tight junction proteins Occludin, in colon tissues (X 100, scale bar, 100μm). Significant results are shown in the box plot. **(D)** Serum D-lactate and diamine oxidase (DAO) levels. *P < 0.05; ***P < 0.001; ****P < 0.0001.

The TEM was utilized to examine the microvilli and tight junctions (TJs) of the colon ultrastructure of mice. Ultrastructurally, microvilli possessed a normal architecture in colonic segments collected from the Sham group, and the TJs immediately beneath the microvilli were narrow and intact. In the CLP and ANC groups, intestinal epithelial cells and intracytoplasmic organelles were significantly enlarged, and microvilli loss was partial. Intercellular space enlargement and mitochondrial enlargement were most pronounced in the ANC group. However, FMT and SCFAs attenuate the destruction of intestinal ultrastructures in CLP-induced sepsis mice (**Figure 4A**).

To test if physiological changes in the TJ barrier were linked to changes in TJ protein distribution, the transmembrane TJ protein Occludin was immunohistochemically labeled in mouse colons. (**Figure 4B**). After 16 hours of CLP, the average integral OD of Occludin in each group was compared. The findings revealed that the expression of Occludin in the ANC group was considerably lower than in the AFC and ASC groups. On the other hand, FMT and SCFAs can augment Occludin expression following CLP.

Signs of a healthy mucus barrier in the stomach include D-lactic acid and DAO. An ELISA was used to measure the amount of D-lactic acid and DAO in each of the five groups and compare them. The results revealed that the Sham group had lower blood concentrations of D-lactic acid and DAO than the CLP and ANC groups. In the AFC group, serum levels of D-lactic acid and DAO were considerably lower than in the ANC group. D-lactic acid and DAO levels did not differ significantly between the ANC and CLP groups (P > 0.05). According to the findings, FMT and SCFAs decrease the increase in intestinal permeability produced by CLP in mice (**Figures 4C, D**).

### 3.5 FMT can restore antibiotic-induced intestinal microbiota disorder

**Figure 5:**
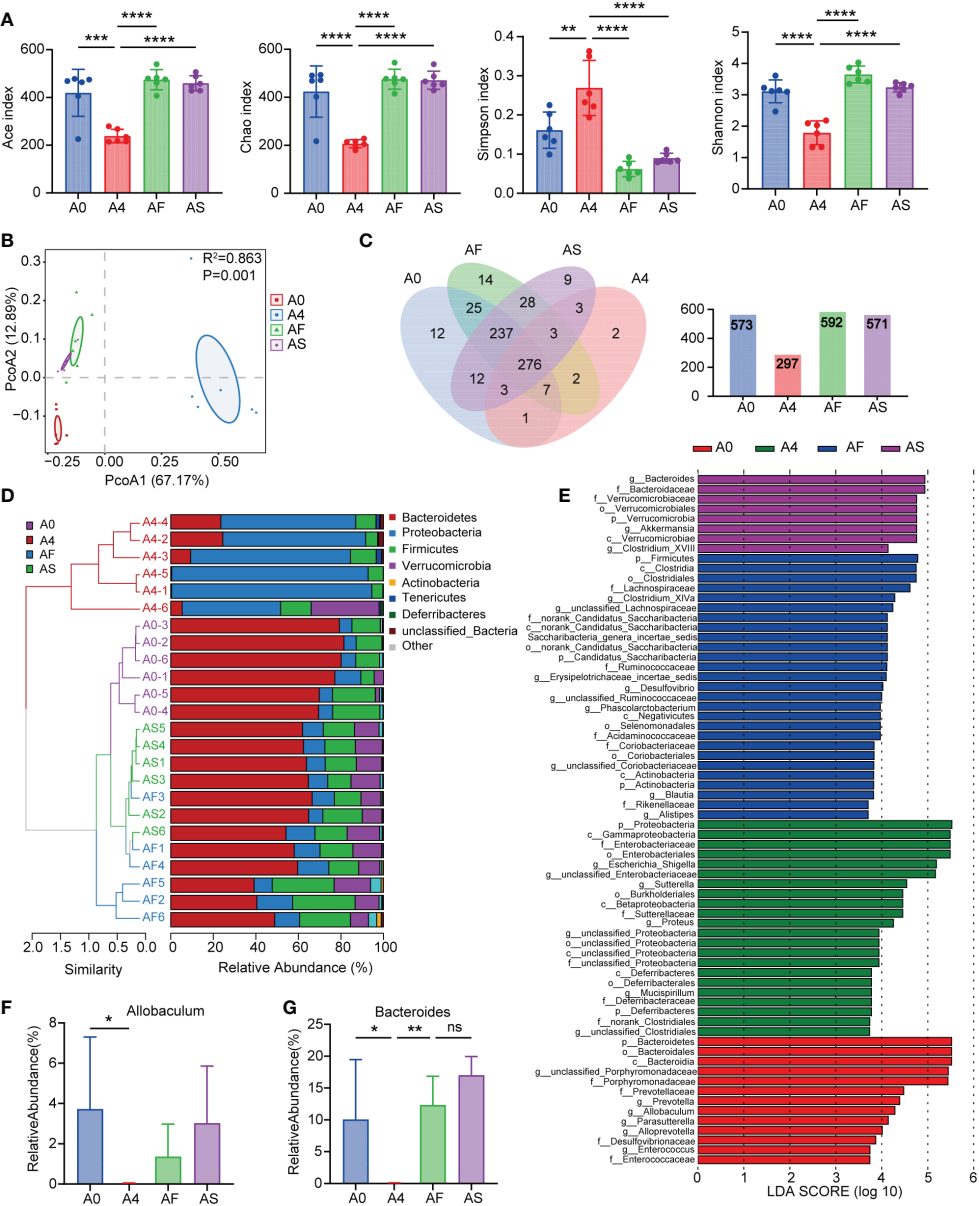
Mouse feces microbiota diversity. **(A)** Alpha diversity in the A0 (blue), A4 (red), AF (green), and AS (purple) groups, as shown by the Ace, Chao, Shannon, and Simpson indices. **(B)** The beta diversity of gut microbiota was displayed by principal component analysis (PCoA) scatterplots. **(C)** Parallel and overlapping circles are seen in this Venn diagram. Species shared between groups are shown by the overlapping portion, while those unique to each group are shown by the non-overlapping portion. **(D)** Hierarchical clustering based on the Weighted Unifrac Distance of OTU profiles shows the community composition of each sample at the phylum level. **(E)** At the genus level, the abundance of species in the LEfSe for each group. The gradation of color represents the correlation value. n = 6 mice per group. **(F, G)** Relative abundance of SCFAs-producing bacteria in groups **(F)** *Allobaculum* **(G)** *Bacteroides*. A0 group (no antibiotics), A4 group (antibiotics for 4 days + normal saline for 3 days), AF group (antibiotics for 4 days + FMT for 3 days), AS group (antibiotics for 4 days + SCFAs for 3 days). *P < 0.05; **P < 0.01; ***P < 0.001; ****P < 0.0001. ns, not significant.

We gavaged three antibiotic mice groups with FMT, SCFAs, and normal saline for three days, respectively, and collected feces to determine whether FMT and SCFAs can regulate the intestinal flora of antibiotic mice. By sequencing the 16S rRNA gene of bacteria, we defined the gut microbiota of animals. The alpha diversity of bacteria (the species richness and diversity of microbiota) was calculated using the Ace, Chao, Shannon, and Simpson indices for a number of populations. Principal coordinates analysis was applied to determine beta diversity (PCoA). Following antibiotic therapy, the diversity and abundance of the microbiota in the intestines of mice decreased. Nonetheless, FMT and SCFAs demonstrated a recovery trend after gavage (**Figures 5A, B**), demonstrating that FMT and SCFAs considerably changed the composition of the gut microbiota in antibiotic-treated mice. The Venn diagram of taxa revealed that there were 573 species in group A0, 297 in group A4, 592 in group AF, and 571 in group AS. After four days of antibiotic treatment, the number of mice’s intestinal flora reduced dramatically (**Figure 5C**). In terms of community abundance composition, the fraction of Bacteroidetes fell and that of Proteobacteria increased after the antibiotic intervention, and the intestinal microbiota of antibiotic-treated mice differed considerably from that of normal mice. However, FMT and SCFAs can repair the abnormal gut microbiota and return it to a more normal state (**Figure 5D**). We filtered the top 50 species in terms of total abundance and evaluated the LEfSe data. *Bacteroides, Prevotella*, *Allobaculum*, *Parasutterella*, *Clostridium-XIVa*, *Alloprevotella*, *Saccharibacteria-genus-incertae-sides*, *Ruminococcaceae*, and *Erysipelotrichaceae-incertae-sedis* decreased after antibiotic therapy. Nonetheless, with FMT and SCFAs gavage, they all recovered. Antibiotic-treated mice (A4 group) displayed a greater number of *Enterobacteriaceae* at the family level (**Figure 5E**). The presence of *Enterobacteriaceae* has been linked to a dismal prognosis in a variety of illnesses (10, 30). However, mice treated with FMT or SCFAs had a reduced abundance of *Enterobacteriaceae*. SCFAs-producing bacteria such as *Allobaculum* (**Figure 5F**) and *Bacteroides* (**Figure 5G**) were considerably less prevalent in the ANC group compared to the AFC and ASC groups.

### 3.6 FMT improved sepsis by inhibiting GSDMD-mediated pyroptosis

**Figure 6:**
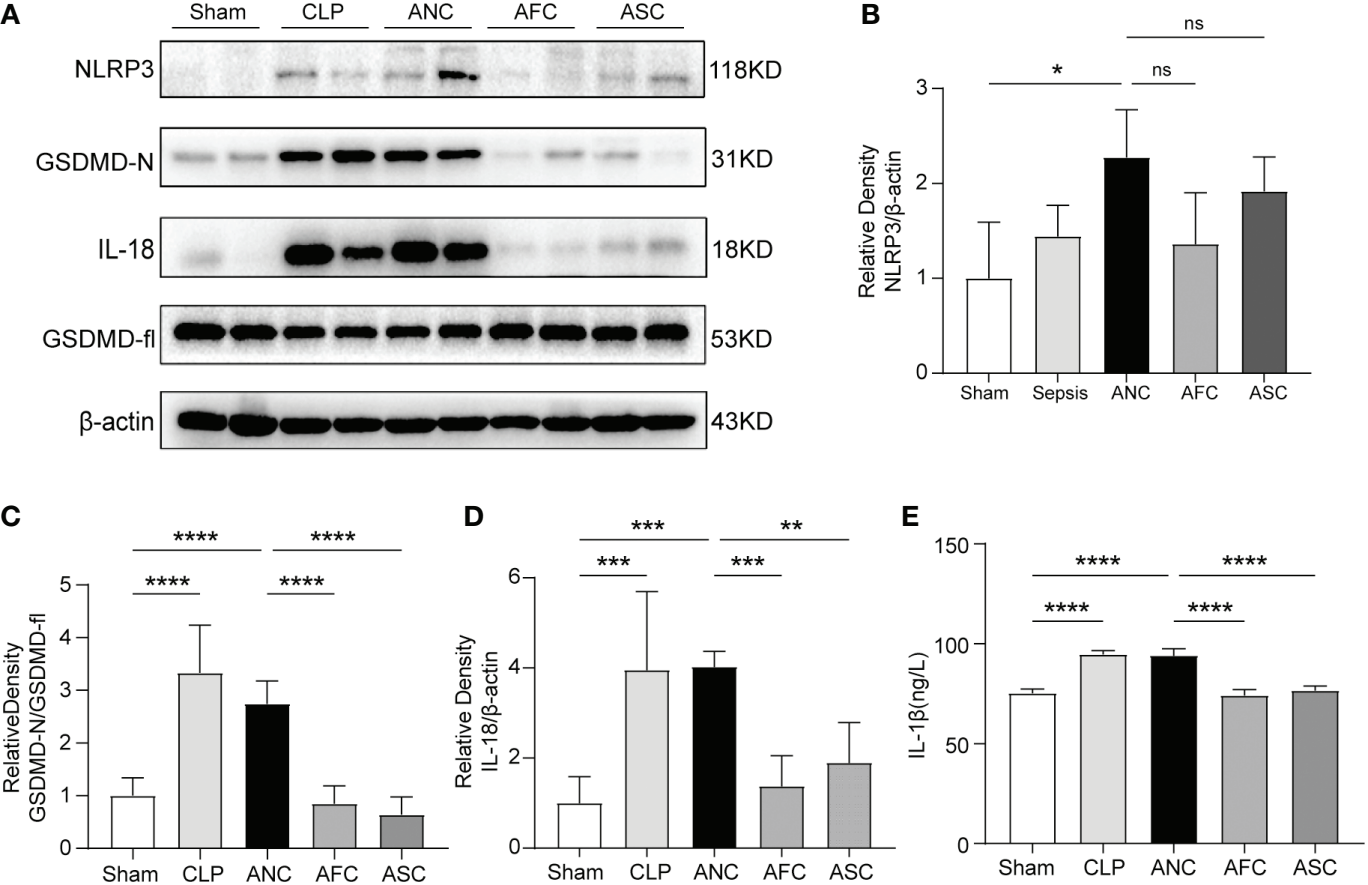
Colonic protein expression of pyroptosis-associated genes after sepsis in mice: the role of Fecal microbiota transplantation (FMT) and short-chain fatty acids (SCFAs). **(A)** Western blotting for the detection of NLRP3, IL-18, GSDMD-fl, and GSDMD-N. **(B–D)** The quantitative examination of the ratio of NLRP3/β-actin, GSDMD-N/GSDMD-fl, and IL-18/β-actin expression was performed, with the Sham group serving as the reference value. **(E)** The serum level of IL-1β was measured using ELISA. n = 6 per group. Data are expressed as the mean ± SD. *P < 0.05; **P < 0.01; ***P < 0.001; ****P < 0.0001; ns: not significant.

Pyroptosis is a type of programmed cell death that is associated with proinflammatory processes and is triggered by caspase-1/11 and gasdermin D. The overactivity of pyroptosis might result in harm to organs. Pyroptosis serves as a defense mechanism for the host against bacterial colonization. In this study, we used western blotting and ELISA to analyze the expression of NLRP3 inflammasome-related and pyroptosis-related proteins in the colon tissues of mice with sepsis. Western blotting revealed that sepsis promoted the expression of NLRP3, IL-18, and GSDMD-N in color tissues, whereas FMT and SCFAs treatment prevented the CLP-induced expression of these proteins (**Figures 6A–D**). In the CLP and ANC groups, serum levels of the proinflammatory cytokine IL-1β increased considerably. FMT and SCFAs reduced blood levels of IL-1β in mice with inflammation induced by CLP (P < 0.001; **Figure 6E**). These findings suggest that FMT and SCFAs administration suppressed the CLP-induced activation of NLRP3 inflammasome and pyroptosis in colon tissues.

### 3.7 Analysis of the fecal metabolism results in mice

**Figure 7:**
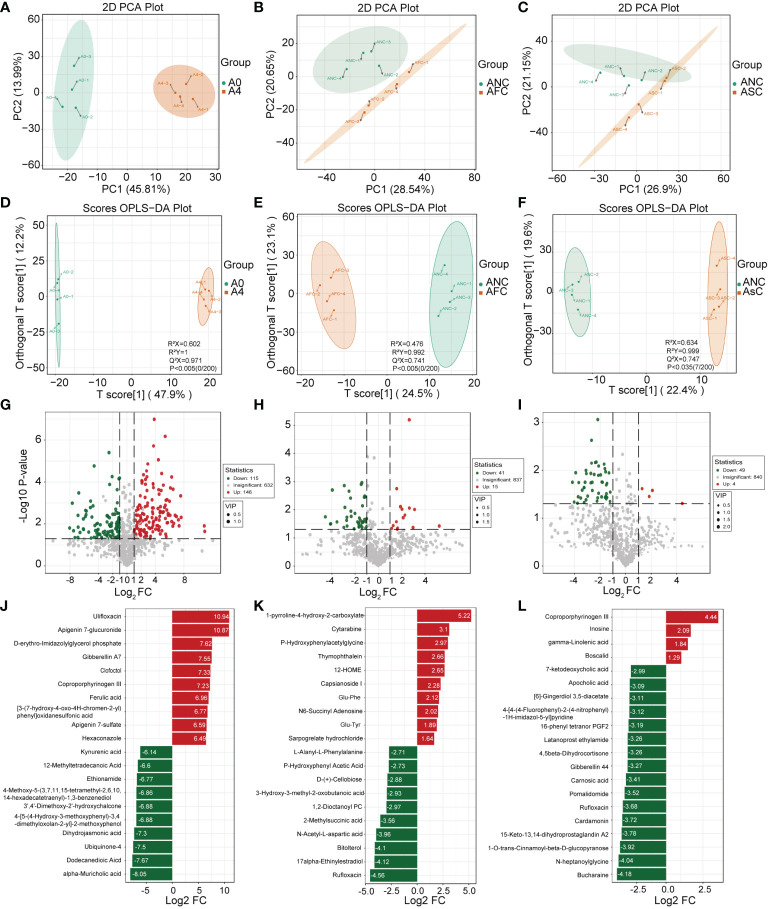
The role of fecal microbiota transplantation (FMT) and short-chain fatty acids (SCFAs) in fecal metabolism in mice with sepsis. **(A–C)** The Scatter plot of principal components analysis (PCA) for metabolites shows that the A0 group had a very different metabolic profile than the A4 group. **(D–F)** OPLS-DA analyses of the metabolite profile. OPLS-DA scores plot and OPLS-DA model test chart showed good discrimination between the groups. Volcano Plot showing differential metabolites between the A0 and A4 groups **(G)** and between the ANC and AFC groups **(H)**, and between the ANC and ASC groups **(I)**. Linear discriminate analysis (LDA) effect size analysis of the top 20 differential metabolites between the A0 and A4 groups **(J)** and between the ANC and AFC groups **(K)**, and between the ANC and ASC groups **(L)**. LDA scores > 1 and significance of p < 0.05 as determined by Wilcoxon’s signed-rank test.

LC-MS analyzed metabolite profiles of the fecal samples. The variation of PC1 and PC2 in the A0 and A4 groups were 45.81% and 13.99%, respectively (**Figure 7A**). In addition, significant variations in metabolite diversity were detected amongst the ANC, AFC, and ASC groups (**Figures 7B, C**). Next, compounds leading to differences among different groups were further analyzed by the OPLS-DA model (31). The scores showed that each group was distributed in two different areas (**Figures 7D–F**). The OPLS-DA model has an excellent fit and good predictive power, as measured by the goodness of fit and predictive ability values.

**Figure 8:**
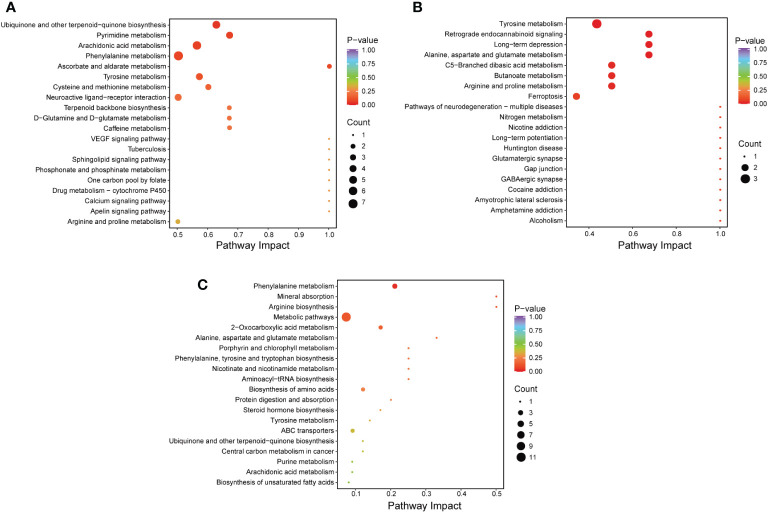
**(A–C)** Comparison of the metabolic pathways of the A0 and A4 groups **(A)**, the ANC and AFC groups **(B)**, and the ANC and ASC groups **(C)**.

In this study, the importance load assessment parameter (VIP), a critical predictor variable, was used as the threshold to further screen for metabolites with level differences between treatment groups, namely differential metabolites (32). Differential metabolites were those that met the following criteria: VIP ≥ 1.0, fold change ≥ 2 or ≤ 0.5, and p < 0.05 (**Figures 7G–L**). 56 differentially metabolites were identified between ANC and AFC groups, and 53 differentially metabolites were identified between ANC and ASC groups. The AFC group showed elevated levels of 41 metabolites and decreased levels of 15 metabolites compared to the ANC group. The ASC group had increased levels of 49 metabolites and reduced 4 metabolites. We utilized the Kyoto Encyclopedia of Genes and Genomes (KEGG) database to build and evaluate metabolic pathways, and we incorporated differential metabolites to further elucidate the critical metabolic pathways between antibiotics and septic gut microbiota. Based on the findings, these metabolites are indicative of critical metabolic pathways such as those involving phenylalanine, arachidonic acid, pyrimidine, tyrosine, cysteine, methionine, alanine, aspartate, glutamate, butanoate, arginine, and proline, and ferroptosis (**Figures 8A–C**).

## 4 Discussion

Sepsis, a potentially fatal dysfunction of the organs, is a major public health issue (33). The importance of gut microbiota in the development of sepsis and organ failure is becoming more and more clear (11). However, in the clinic, sepsis organ failure is evaluated primarily in the pulmonary, cardiovascular, hepatic, renal, nervous, and hematologic systems. Due to a lack of diagnostic specificity, gastrointestinal symptoms were not included in the analysis (34). Therefore, it is crucial for the development of microbial targeted therapy to have a comprehensive understanding of the changes in the microbiome in sepsis.

In this study, we employed 16S rRNA sequencing to evaluate fully the effects of sepsis on the human gut microbiota. Our results show that sepsis has a major impact on the gut microbiota and that intestinal dysbiosis is also crucial to the development of organ damage during sepsis. The NMDS analysis showed that the microbial communities of people with sepsis were very different from those of healthy people and were located in different quadrants. Also, sepsis patients had a lot less microbiome diversity, which was in line with a previous study (35). Sepsis patients have decreased levels of beneficial bacteria, while the quantity of pathogenic bacteria grows dramatically. At the phylum level, the amount of Proteobacteria in the intestinal flora of sepsis patients was much larger than that of the control group, whereas the number of Firmicutes was significantly lower. Additionally, *Roseburia*, *Blautia*, *Clostridium*, and *Anaerostipes* are SCFAs producers and are among the severely diminished genus (36). Remarkably, we observed that sepsis patients had a dramatically higher abundance of gut *Enterococcus* compared to healthy controls. It has been found that when SCFAs-producing bacteria are depleted, vancomycin-resistant *Enterococcus* populations grow in critically ill patients (37). Reduction in butyrate, an effector chemical controlling anti-inflammatory signals and glucose and lipid metabolism, has been linked to the presence of *Enterococcus* species in the colon (38). Our results suggest that gut microbiota dysbiosis in sepsis has the following two distinctive features. First, when sepsis develops, the microbiome’s structure changes, and the microbiota’s richness and diversity diminish. Second, alterations in the gut microbiota may predispose individuals to sepsis by promoting the expansion of potentially harmful bacteria and lowering the generation of SCFAs. Therefore, studying the relationship between sepsis and gut microbes and metabolites is necessary.

Previous research has shown that FMT is effective in treating Clostridioides difficile infections (CDI) in people, which have been linked to the overuse of antibiotics (39). It involves transplanting healthy donors’ functional bacteria into a patient’s gastrointestinal tract to repair the intestinal microecological balance and treat microbial imbalance-related disorders (40). Recent clinical trials have demonstrated that FMT is effective in eradicating colonized drug-resistant bacteria in patients with hematological malignancies and restoring treatment for colitis (41, 42). In addition, insulin sensitivity is improved in patients with severe obesity and metabolic syndrome when FMT is combined with a daily intake of low-fermentable fiber (43). The increasing significance of the microbiome in health has prompted more efforts to treat a variety of diseases, including autoimmune disorders, metabolic syndrome, and sepsis (44, 45). Due to the alteration of the microbiome during sepsis, researchers and physicians have turned to FMT as an adjunct to sepsis therapy. The use of FMT to treat patients with therapy-resistant sepsis and diarrhea is described in two case reports (46, 47). However, we do not fully comprehend which bacterial species would engraft following FMT, nor do we have a suitable approach for screening donor samples for potentially hazardous bacteria. Therefore, we need more extensive prospective studies and animal experiments to confirm the role of FMT in sepsis. Similarly, studies have shown intestinal flora metabolite SCFAs can significantly affect the immune system, leading to dysregulation of processes such as sepsis and correction during flora repair (19). Maintaining a healthy gut flora and normal amounts of SCFAs results in a more tolerant immune system.

As more and more is learned about the role of the microbiota in sepsis, the balance will shift in favor of non-traditional therapies. In patients at risk of dysbiosis, a tailored microbiota therapy that involves the replacement of particular commensal or short-chain fatty acids-producing bacteria may reduce their susceptibility to sepsis (48, 49). Based on these findings, relevant animal tests were done. We postulated that FMT or SCFAs could mitigate dysbiosis, promote gut microbial barrier mending, and ultimately contribute to the treatment of sepsis. These results indicated that antibiotic use might change the species composition and diversity of gut microbiota. Mice with gut microbiota disorders (ANC group) were found to have an elevated risk of death, inflammation, and organ failure as compared to CLP mice. Mortality, histological examination, and inflammatory cytokine levels all demonstrated that the sepsis model could be activated, and that it resulted in severe pathological damage. At the same time, all of these could be reversed by FMT and SCFAs. The impact of sepsis on organs can extend the related microbiome changes. Targeting the gut microbiota as a treatment for sepsis can minimize inflammatory damage and preserve organ function. Maintaining the integrity of the mucosal epithelium is dependent on tight connections (50, 51). We compared the results of transmission electron microscopy among the five groups. We found that the TJs of the ANC group were more blurred than those of the AFC and ASC groups, with large villi shedding and swelling of terminal lysosomes and organelles. We hypothesize that the mice exhibited gut barrier and cell malfunction. Occludin’s primary responsibilities are to maintain TJs in place and seal them (52). Next, we used immunohistochemistry to look at the expression of Occludin protein in five groups of colon tissues. The results showed that the amount of Occludin protein in the ANC group was much lower than that in the Sham group. The AFC and ASC groups had higher index scores than the ANC group or scores that were the same as the Sham group.

Pyroptosis is an essential immunological response that plays a crucial role in the development and occurrence of sepsis. During the initial phase of sepsis, the body initiates pyroptosis, which slows the intracellular multiplication of infections and promotes their clearance (53). If the infection is not controlled, several microorganisms will infiltrate the blood and cells in order to evade immune system recognition and removal. During this process, pathogen-related molecular patterns and DAMPs are produced, producing widespread pyroptosis, elevating IL-18 and IL–1β levels, exacerbating the systemic inflammatory response, and finally resulting in organ failure and septic shock (54, 55). GSDMD is a pyroptosis effector molecule. When GSDMD cleavage cytotoxicity is activated and the GSDMD-N-terminal cleavage product localizes to the plasma membrane, holes are formed and IL-1β and IL-18 are released (56, 57). Our result showed a considerable decrease in NLRP3, GSDMD-N, IL-18 protein expression, and serum IL-1β levels in the AFC and ASC groups compared to the ANC group, indicating that FMT and SCFAs can suppress cell pyroptosis and have a protective function in sepsis (**Figures 5A–E**).

This study aimed to determine whether FMT and SCFAs can protect the intestinal barrier function and preserve the intestinal flora in mice with sepsis. First, we investigated the makeup of gut microbiota using high-throughput sequencing of 16S rRNA. After simulating sepsis, we discovered a flora imbalance after four days of antibiotic gavage, and the ANC group had the most serious intestine injury. The relative abundance of Bacteroides and Firmicutes reduced greatly following the administration of antibiotics to mice, but grew dramatically following FMT and SCFAs. Bacteroidetes and Firmicutes are the primary bacterial species that colonize the large intestine and ferment dietary fiber; they are the leading producers of butyrate, which has been verified, and contribute to intestinal health (58). Moreover, the relative abundance of Proteobacteria increased dramatically following antibiotic gavage in mice, but reduced significantly following FMT and SCFAs. Gram-negative Proteobacteria include numerous harmful and lipopolysaccharide-containing bacteria, such as *Escherichia coli*. According to the sequencing results on the fecal microbial diversity of mice, the relative abundance of *Escherichia-Shigella* in the stomach considerably increased after antibiotics were gavaged into the animals. *Escherichia-Shigella* is one of the most prevalent intestine pathogenic bacteria, characterized by colonic epithelial invasion and destruction. An increased number of gut *Enterobacteriaceae* is regarded as a factor that is unfavorable to the patient’s prognosis (10). It has been suggested that an increase in the number of Enterobacteriaceae in the gut may contribute to the worsening of stroke patients’ symptoms, including brain infarction and systemic inflammation (30). Additionally, gut *Enterobacteriaceae* overgrowth increases sepsis and death risk (59). The results of our study demonstrated that FMT and SCFAs not only increased the abundance and diversity of the microbiota of antibiotic-treated mice, but also significantly reduced the number of *Enterobacteriaceae*, suggesting that FMT and SCFAs ameliorates mortality from sepsis by inhibiting the overgrowth of *Enterobacteriaceae* in the gut. Among the AS group, an increase in the genus *Akkermansia* was found; this particular strain has shown promise as a probiotic in animal and human studies (53, 54). According to a study, *Akkermansia* can promote gut health by sticking to the intestinal epithelium and enhancing the monolayer integrity of enterocytes (56). Therefore, we recommend that SCFAs may play a protective role against sepsis by increasing the number of *Akkermansia*. Another study has shown that butyrate-producing bacterias such *Ruminococcaceae*, *Faecalibacterium*, and *Roseburia* are reduced in ulcerative colitis (60). Therefore, the relative abundance of *Ruminococcaceae* was compared in this experiment. The results revealed a considerable drop in the antibiotic group, whereas the fecal bacteria transplantation and short-chain fatty acid groups had outcomes comparable to those of normal mice. It is clear that *Ruminococcaceae* plays a role in the inflammatory response, and that FMT and SCFAs can effectively modify the composition of the bacteria, thereby alleviating the inflammatory state. Our investigation demonstrated that FMT and SCFAs regulated the abundance of bacteria such as Firmicutes, Proteobacteria, *Escherichia Shigella*, and *Lactobacillus*, restoring them to levels comparable to those of healthy mice, indicating that these bacteria may mediate the positive effects of FMT and SCFAs. FMT and SCFAs may modify the gut microbiota of septic mice by increasing helpful bacteria and lowering harmful bacteria. In conclusion, the results indicate that mice with decreased gut microbiota had greater bacterial dispersion, inflammation, organ failure, and premature mortality in comparison to the CLP group.

At the same time, non-targeted metabolome analysis was performed on feces samples from mice. The results of our investigation indicate that antibiotic therapy dramatically modifies the metabolic profile of feces and affects the outcome of sepsis. The major implicated metabolic pathways are tyrosine metabolism, arachidonic acid metabolism, cysteine and methionine metabolism, alanine, aspartate and glutamate metabolism, butanoate metabolism, arginine and proline metabolism, and ferroptosis. Amino acid metabolism abnormalities have been observed to be widespread in wasting disorders like sepsis (61). In addition, Huang et al. discovered that elevated phenylalanine levels in sepsis are associated with a worse prognosis (62). In this study, we also discovered that the phenylalanine level in the ANC group was much higher than in the AFC and ASC groups, suggesting that it may be associated to the poor prognosis of ANC mice.

## 5 Conclusion

**Figure 9:**
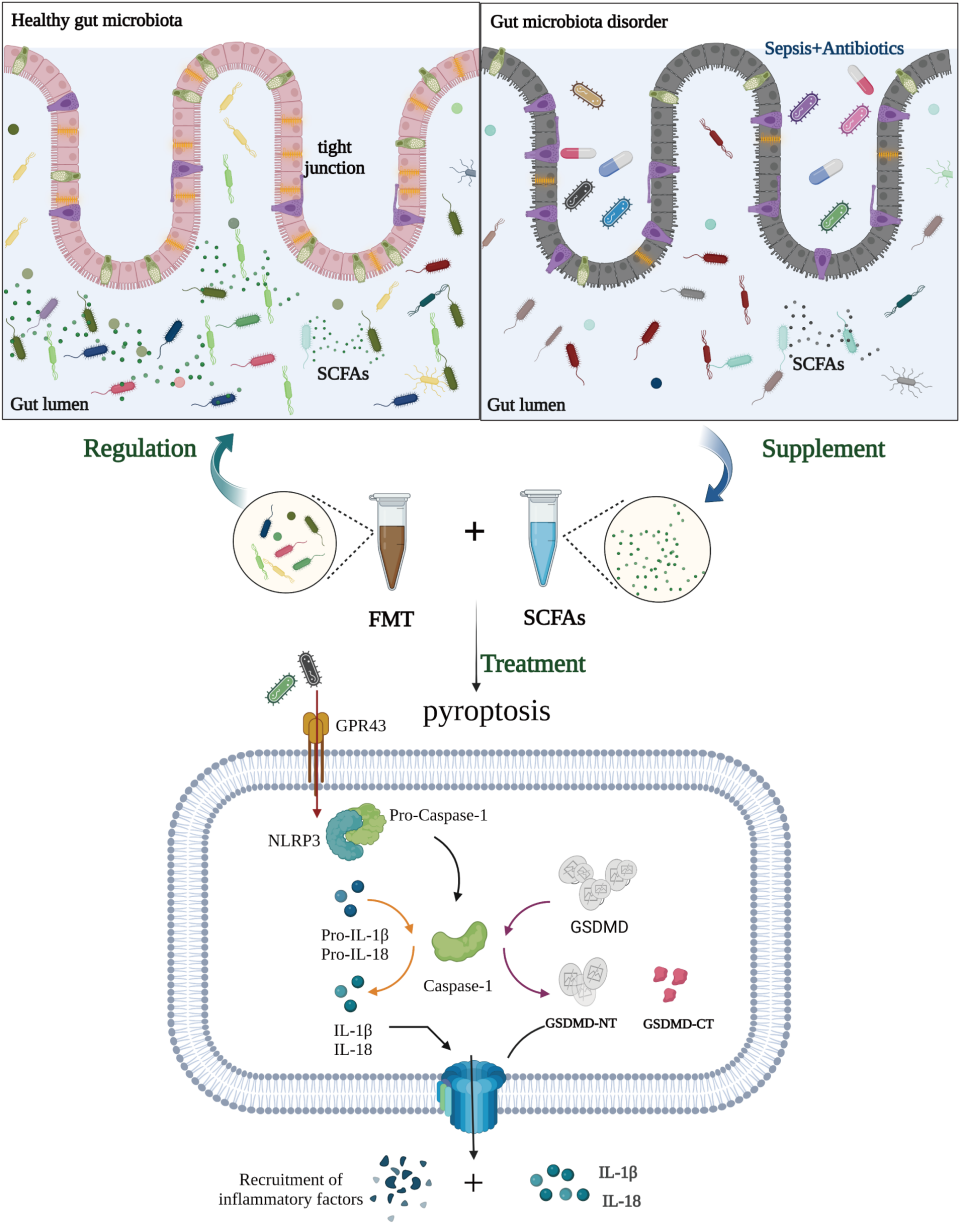
Potential role of fecal microbiota transplantation (FMT) and short-chain fatty acids (SCFAs) in treating sepsis. In the pathogenesis of sepsis, there is an imbalance in the intestinal flora, which decreases the production of SCFAs from intestinal flora metabolites and decreases the ability of SCFAs to bind to GPR43. It also induces further NOD-like receptor protein-3 (NLRP3) inflammasomes to be made and more IL-1β and IL-18 to be released, exacerbating the inflammatory response of tissues and cells. In addition, NLRP3 triggers the activation of caspase-1, which in turn cleaves the gasdermin D (GSDMD) protein and triggers cell pyroptosis, hence exacerbating sepsis and perpetuating the vicious cycle. FMT and SCFAs can protect intestinal function, regulate the distribution and amount of intestinal flora, restore dominant intestinal flora, boost the production of SCFAs, limit inflammatory response and cell pyroptosis, and subsequently protect against sepsis. (Created with BioRender.com).

In conclusion, we discovered dysbiosis of the intestinal microbiota in sepsis patients. FMT and SCFAs dramatically reduced the mortality of septic mice treated with antibiotics, as well as intestinal damage and intestinal flora disturbance. In addition, FMT increased the expression of the Occludin protein in the colon of mice with sepsis, downregulated the expression of the NLRP3 and GSDMD-N proteins, and reduced the release of the inflammatory factors IL-1β and IL-18 to inhibit cell pyroptosis, ultimately playing a protective role in sepsis. FMT and SCFAs are feasible and effective adjunctive therapy options for sepsis. Early treatment with FMT or SCFAs can greatly reduce mortality in mice with sepsis, most likely as a result of the restoration of gut flora (**Figure 9**). Our results may contribute to a better understanding of the function of the gut microbiome in the etiology of sepsis and to the development of preventive measures based on the manipulation of the gut microbiome.

## Data availability statement

The data presented in the study are deposited in the NCBI repository, accession number PRJNA891660.

## Ethics statement

The experiments were approved by the First People’s Hospital of Yunnan Province medical ethics committee (Project No: KHLL2021-036). The patients/participants provided their written informed consent to participate in this study. This study was conducted according to the National Institutes of Health guidelines and was approved by the Research Ethics Committee of Kunming Medical University (Project No: KMMU20221600).

## Author contributions

XL and JX were responsible for the design and preparation of the study and drafted the manuscript. GC was in charge of supervising the experiments and revising the manuscript draft. RS and YY conducted the experiments. DN, CM, and FW analyzed the data, and calculated the figures. All authors contributed to the article and approved the submitted version.
